# Pedigree investigation, clinical characteristics, and prognosis analysis of haematological disease patients with germline *TET2* mutation

**DOI:** 10.1186/s12885-022-09347-0

**Published:** 2022-03-12

**Authors:** Xia Wu, Jili Deng, Nanchen Zhang, Xiaoyan Liu, Xue Zheng, Tianyou Yan, Wu Ye, Yuping Gong

**Affiliations:** Department of Haematology, West China Hospital, Sichuan University, No.37 GuoXue Xiang, Chengdu, 610041 Sichuan Province China

**Keywords:** Germline *TET2* mutation, Somatic *TET2* mutation, Myelodysplastic syndromes, Acute myeloid leukemia

## Abstract

**Background:**

Increasing germline gene mutations have been discovered in haematological malignancies with the development of next-generation sequencing (NGS), which is critical for proper clinical management and long-term follow-up of affected individuals. Tet methylcytosine dioxygenase 2 (*TET2*) is one of the most common mutations in haematological neoplasms. We aimed to compare the clinical characteristics of patients with germline and somatic *TET2* mutations in haematological diseases and to analyse whether germline *TET2* mutations have a family aggregation and tumour predisposition.

**Methods:**

Out of 612 patients who underwent NGS of 34 recurrently mutated genes in haematological diseases, 100 haematological patients with *TET2* mutations were selected for further study. Somatic mutations were detected by NGS in bone marrow/peripheral blood genomic DNA (gDNA). Germline *TET2* mutations were validated in nail/hair gDNA by Sanger sequencing. Digital data were extracted from the haematology department of the West China Hospital of Sichuan University. *TET2* mutation results were analysed by referencing online public databases (COSMIC and ClinVar).

**Results:**

One hundred patients were studied, including 33 patients with germline and 67 patients with somatic *TET2* mutations. For germline *TET2* mutations, the variant allele frequency (VAF) was more stable (50.58% [40.5–55], *P* < 0.0001), and mutation sites recurrently occurred in three sites, unlike somatic *TET2* mutations. Patients with germline *TET2* mutations were younger (median age 48, 16–82 years) (*P* = 0.0058) and mainly suffered from myelodysplastic syndromes (MDS) (*n* = 13, 39.4%), while patients with somatic *TET2* mutations were mainly affected by acute myeloid leukemia (AML) (*n* = 26, 38.8%) (*P* = 0.0004). Germline *TET2* mutation affected the distribution of cell counts in the peripheral blood and bone marrow (*P* < 0.05); it was a poor prognostic factor for MDS patients via univariate analysis (HR = 5.3, 95% CI: 0.89–32.2, *P* = 0.0209) but not in multivariate analysis using the Cox regression model (*P* = 0.062).

**Conclusions:**

Germline *TET2* mutation might have a family aggregation, and *TET2* may be a predisposition gene for haematological malignancy under the other gene mutations as the second hit. Germline *TET2* mutation may play a role in the proportion of blood and bone marrow cells and, most importantly, may be an adverse factor for MDS patients.

**Supplementary Information:**

The online version contains supplementary material available at 10.1186/s12885-022-09347-0.

## Background

The role of germline gene mutations in tumours has been increasingly recognised since the occurrence and wide application of next-generation sequencing (NGS), especially in haematological neoplasms [1]. Woo-Joo Song et al. first reported that germline *RUNX1* mutation was associated with familial platelet disorder predisposition to acute myeloid leukemia (FPD/AML) [2]. However, owing to the difficulty in collecting samples of germline DNA specimens, the development and exploration of germline gene mutations in haematological diseases is relatively slow compared with solid tumours. Although skin fibroblasts are the gold standard of specimens for germline mutation testing [3], hairs/nails have recently been reported to be a reliable source of germline DNA [1, 4, 5], which largely contributes to the recognition of germline mutations in haematological diseases. For instance, germline *CEBPA* and *DDX41* mutations are related to the family predisposition to myelodysplastic syndrome (MDS) and AML [6–8]. Germline *RUNX1* mutation is associated with family inherited platelet disease and a high risk of transformation to MDS/AML [2, 9], and germline *GATA2*, *ANKRD26*, and *ETV6* mutations are reported to be related to genetically heritable haematological malignancies (HMs) [10–12]. Therefore, the 2016 World Health Organisation (WHO) classification proposed a new and distinct entity of myeloid neoplasms with germline predisposition [13]. Considering this significance, we conducted an investigation on haematological patients with germline mutations in the past year (2020) and found that among 209 patients with haematological diseases, 33 (15.8%) patients had germline Tet methylcytosine dioxygenase (*TET2*) mutation, which was the second most common mutation after *ZRSR2* (17.7%) (unpublished observations).

*TET2*, a gene involved in DNA demethylation, mainly catalyses the conversion of 5-methylcytosine (5-mC) to 5-hydroxymethylcytosine (5-hmC) to contribute to DNA demethylation [14]. *TET2* is highly expressed in hematopoietic stem cells (HSCs) and significantly affects the self-renewal, differentiation, and proliferation of HSCs [15]. *TET2* mutations are common in haematological neoplasms and can occur in 30% of myelodysplastic syndrome (MDS), 20% of myeloproliferative neoplasms (MPNs), 30% of secondary acute myeloid leukemia (sAML), 17% of novel AML, and 50–60% of chronic myelomonocytic leukemia (CMML) cases [16–19]. Furthermore, many studies on DNA demethylation agents, such as decitabine (DAC) or 5-azacitidine (AZA), have been reported, especially in haematological myeloid neoplasms [20–22].

However, germline mutations in *TET2* have not been reported thus far. Considering the updated 2016 WHO classification, the significant role of *TET2* mutation in haematological neoplasms, and data from our previous research, we performed this study mainly to understand if germline *TET2* mutation has a family aggregation phenomenon and is a tumour predisposition gene. Furthermore, we compared its impact in haematological diseases with somatic *TET2* mutations, including their mutation sites, variant allele frequency (VAF), diagnosis distributions, blood cell counts, prognosis, and survival.

## Methods

### Patients and samples collection

We received approval from the ethics committee of West China Hospital of Sichuan University, China, and obtained informed consent from all patients and their family members in accordance with the Declaration of Helsinki.

**Fig. 1:**
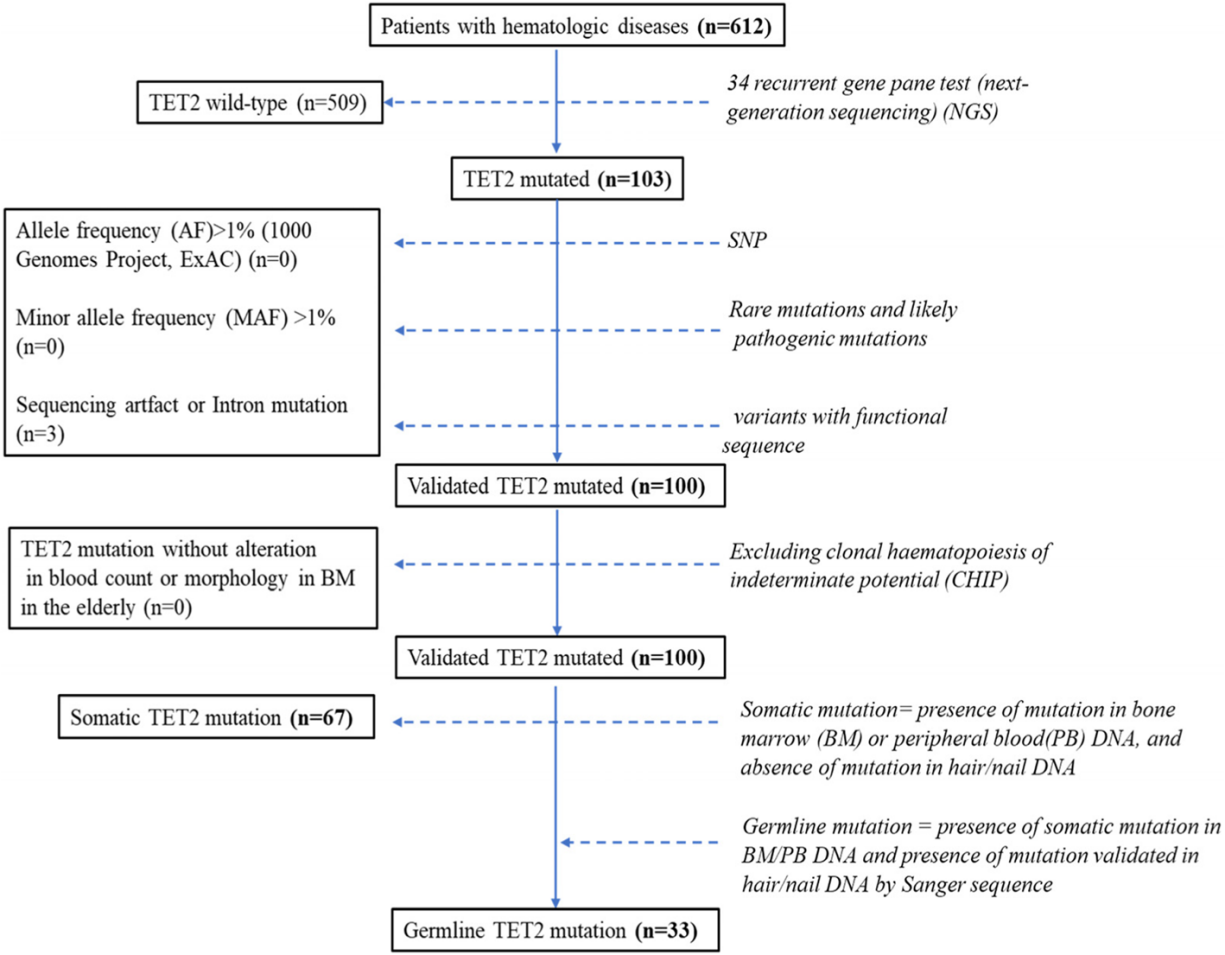
Pipeline used for the identification of germline and somatic *TET2* mutations

A total of 100 patients with *TET2* mutations were selected from 612 patients who underwent the 34 myeloid gene panel test (S1 Table) in our institution (West China Hospital of Sichuan University) from December 2016 to December 2019, including 33 (33%) patients with germline mutations and 67 (67%) patients with somatic mutations (Fig. 1). Fresh specimens were collected at diagnosis, including bone marrow (BM)/peripheral blood (PB) as somatic DNA origin and nails/hair as germline DNA origin [1, 4, 23] to extract genomic DNA (gDNA) and carry out next-generation sequencing (NGS) and Sanger sequencing, respectively. By referencing the allele frequency (AF) in population databases (1000 Genomes Project, ExAC), we removed mutations with more than 1% AF with the possibility of a single nucleotide polymorphism (SNP) [24, 25]. If the minor allele frequency (MAF) is less than 1% or is not reported in the two public databases, it will be considered a rare variate and will be included in the analysis [26]. Mutation results were referenced to the Human Reference SNP (rs) Report and gnomAD database (S2 Table) to remove the sequencing artifacts and restrict variants with functional sequences, such as nonsense, missense, frameshift, etc. Because somatic *TET2* mutations are usually associated with clonal haematopoiesis of indeterminate potential (CHIP) [27, 28], we excluded old patients (> 65 years) with *TET2* mutations who had normal peripheral blood (PB) cell counts or morphology in the bone marrow (BM). Finally, if somatic mutations were detected in BM/PB by NGS, Sanger sequencing in hair/nails was performed to verify whether they were germline mutations (Fig. 1). If the DNA of the nail/hair is not found to have the same mutation as that of the BM/PB, the mutation is identified as a somatic mutation; otherwise, it is a germline mutation. The hair/nails of family members of proband patients with germline *TET2* mutation and as many peripheral blood samples as possible were collected to detect the *TET2* status. The patients were diagnosed by haematologists by combining morphology, cytogenetics, immunophenotyping, and molecular genetics (MICM) according to the 2008 and 2016 WHO guidelines. Only patients who accepted therapies were included in the survival analysis.

### Sequence

The collected samples were sent to the Hubei Wuhan Kindstar Global Esoteric Test Service Work. For somatic mutations, gDNA extracted from fresh BM/PB was used to complete NGS covering 34 targeted genes (34 Myeloid Panel, 34-MP) (S1 Table) by multiplex polymerase chain reaction (PCR) using Ion Ampliseq technology. The constructed libraries were tested using the Ion Proton semiconductor of the Life Technologies platform. For germline mutations, gDNA extracted from hair/nails was used for Sanger sequencing. The sequencing depth was at least 500 reads. The *TET2* detection area was mainly from exons 3 to 11 for the patients.

Disease databases (Catalogue of Somatic Mutations in Cancer (COSMIC) and ClinVar) were referenced for analysing the clinical significance of mutations. We mainly checked germline *TET2* mutation sites in ClinVar (https://www.ncbi.nlm.nih.gov/clinvar) and somatic *TET2* mutation sites in COSMIC (http://cancer.sanger.ac.uk/cosmic/gene/analysis?ln=*TET2*) to determine whether there are relative clinical reports of these mutation sites.

### Statistical analyses

Statistical calculations of materials were performed using SPSS version 24.0 and GraphPad Prism 7. The value of *P* is two-tailed, and less than 0.05 was considered statistically significant. All the tests were two-sided. Categorical variables are described as counts and relative frequencies (percentages), and continuous variables are described as medians and ranges. Comparisons of categorical variables were performed by Fisher’s exact test or the X^2^ test, and continuous variables were compared by the Mann–Whitney’s U test. Survival analyses were calculated by the Kaplan–Meier test for univariate analyses (log-rank test). A Cox proportional hazard model for multivariate analyses. OS was based on death from any cause.

## Results

### The basic characteristics of the patients with germline and somatic TET2 mutations

As shown in Table 1, we included 33 patients with germline *TET2* mutations, including 18 men (54.5%) and 15 women (45.5%). The median age of all patients was 48 years (range, 16–82). The median VAF of germline *TET2* mutations was 50.58% (range, 40–55%). The most common mutation was a missense mutation (90.9%). Eleven mutation sites were detected, mainly in exons 3 to 11. Among them, nine mutation sites were specifically located in exon 3, one in exon 6, and one in exon 10. Furthermore, three mutation sites were highly recurrent: c.2604 T > G (p. Phe868Leu), c.3116C > T (p. Ser1039Leu), and c.2440C > T (p. Arg814Cys). The frequencies of these three sites were 12 (36.36%), 9 (27.27%), and 4 (12.12%), respectively, while the others occurred only once. The AF of six mutation sites was less than 1% in the 1000 Genomes and ExAC databases, and the AF of five mutation sites was not reported in the two databases. The recurrent mutation sites were also detected in the ClinVar database (S3 Table). Among the 33 patients, 18 (54.5%) had a normal chromosomal karyotype, two (6.1%) had complex karyotypes, six (18.2%) had other abnormal karyotypes, and seven (21.2%) had no available karyotype results. Summarising the distribution of disease in 33 patients, we found that 13 (39.4%) patients had MDS, four (12.1%) had aplastic anaemia (AA), three (9.1%) had AML, five (15.1%) were diagnosed with other diseases, and eight (21.2%) were undiagnosed (Table 1). We then classified the MDS according to the 2008 and 2016 WHO guidelines and AML according to the FAB guidelines (Table 1).

**Table 1:** Clinical characteristics of patients with *TET2* gene germline mutations

Patient No.	Gender	Diagnosis	VAF	Mutation type	Mutation site	Exon	Karyotype	MAF	1000 genome	ExAC
**Age (<=60 years)**										
1	M	Neutropenia	0.5234	missense	c.2604 T > G(p.Phe868Leu)	3	46,XY [20]	0.0024	0.0024	0.00233
2	F	?	0.5048	missense	c.2604 T > G(p.Phe868Leu)	3	46,XY [20]	0.0024	0.0024	0.00233
3	F	?	0.493	missense	c.455G > A(p.Ser152Asn)	3	NA	NR	NR	NR
4	M	MDS-U	0.5258	missense	c.2604 T > G(p.Phe868Leu)	3	46,XX [20],+(8), UPD(11p)	0.0024	0.0024	0.00233
5	M	?	0.509	missense	c.3116C > T(p.Ser1039Leu)	3	NA	0.0012	0.0012	0.00161
6	M	CML	0.5416	missense	c.2604 T > G(p.Phe868Leu)	3	NA	0.0024	0.0024	0.00233
7	F	AA	0.5285	missense	c.3116C > T(p.Ser1039Leu)	3	46,XX [20]	0.0012	0.0012	0.00161
8	M	?	0.4902	missense	c.2604 T > G(p.Phe868Leu)	3	NA	0.0024	0.0024	0.00233
9	M	HC	0.502	missense	c.3116C > T(p.Ser1039Leu)	3	NA	0.0012	0.0012	0.00161
10	F	AA	0.511	missense	c.3116C > T(p.Ser1039Leu)	3	46,XY [20]	0.0012	0.0012	0.00161
11	F	AML-M2	0.475	missense	c.3728A > G(p.Lys1243Arg)	6	46,XX,t(8;21)(q22;q22)	NR	NR	NR
12	F	?	0.4841	missense	c.3116C > T(p.Ser1039Leu)	3	NA	0.0012	0.0012	0.00161
13	M	AA	0.482	missense	c.3106C > T(p.His1036Tyr)	3	46,XY [15]	NR	NR	NR
14	F	IDA	0.5224	missense	c.2604 T > G(p.Phe868Leu)	3	46,XY [20]	0.0024	0.0024	0.00233
15	M	MDS-U	0.5094	missense	c.2604 T > G(p.Phe868Leu)	3	46,XY [20]	0.0024	0.0024	0.00233
16	F	AML-M4	0.4867	missense	c.2440C > T(p.Arg814Cys)	3	46,XY [20]	0.0014	0.0014	0.0006
17	F	MDS-SLD	0.5023	missense	c.5816A > G(p.Tyr1939Cys)	3	46,XX [20]	NR	NR	NR
18	M	?	0.512	missense	c.1712G > A(p.Arg571His)	3	NA	NR	NR	0.00003
19	M	MDS-EB2	0.4984	missense	c.2604 T > G(p.Phe868Leu	3	44 ~ 45,XY,-6,-7,+ 13,-17,-21,-22 +r,+ 3-, 4mar,inc[cp4]/46,XY [1]	0.0024	0.0024	0.00233
20	M	MDS-SLD	0.5332	missense	c.3116C > T(p.Ser1039Leu)	3	46,XY [20]	0.0012	0.0012	0.00161
21	F	AML-M2	0.5253	missense	c.2604 T > G(p.Phe868Leu)	3	46,XX, t(6;11)(q27;q23) [19]/46,xx [1]	0.0024	0.0024	0.00233
22	F	MDS-EB2	0.5061	missense	c.427G > A(p.Asp143Asn)	3	46,XX [20]	NR	NR	0.00003
**Age (> 60 years)**										
23	F	MDS-RA	0.5058	missense	c.2440C > T(p.Arg814Cys)	3	46,XX [20]	0.0014	0.0014	0.0006
24	F	MDS-SLD	0.4912	missense	c.2440C > T(p.Arg814Cys)	3	47,XX,+add(1)(p11.2) [20]	0.0014	0.0014	0.0006
25	F	AA	0.5078	missense	c.2440C > T(p.Arg814Cys)	3	46,XY [20]	0.0014	0.0014	0.0006
26	F	MDS-U	0.4835	missense	c.218G > A(p.Arg73His)	3	46,XX,-20,+mar [20]	NR	NR	0.00001
27	M	MDS-EB2	0.55	missense	c.2604 T > G(p.Phe868Leu)	3	46,XY,der(7)t(1;7) (q10;p10) [20]	0.0024	0.0024	0.00233
28	M	MDS-MLD	0.5125	missense	c.3116C > T(p.Ser1039Leu)	3	46,XX [20]	0.0012	0.0012	0.00161
29	M	?	0.4965	missense	c.3116C > T(p.Ser1039Leu)	3	46,XY [20]	0.0012	0.0012	0.00161
30	M	MDS-RAEB1	0.4845	missense	c.4183G > A(p.Val1395Ile)	10	46 ~ 48,XY,+ 1,-5,del(5) (q13q33),+ 8,-9,-18,-20, + 2 ~ 4mar1,+mar2[cp20]	NR	NR	NR
31	M	MDS-RAEB2	0.5259	missense	c.2604 T > G(p.Phe868Leu)	3	46,XX [20]	0.0024	0.0024	0.00233
32	M	ITP	0.4985	missense	c.3116C > T(p.Ser1039Leu)	3	46,XY [20]	0.0012	0.0012	0.00161
33	M	?	0.5002	missense	c.2604 T > G(p.Phe868Leu)	3	46,XX [13]	0.0024	0.0024	0.00233

Abbreviation: *Patient No *patient’s number; *VAF* variate allele frequency; *MAF* minor allele frequency; *ExAC* Exome Aggregation Consortium; *M* male; *F* female; *HC* hepatic cirrhosis; *IDA* iron deficiency anemia; *NA* no available; *NR* no report;?, undiagnosed; *AML* acute myeloid leukemia; *MDS* myelodysplastic syndrome; *MDS-RA* MDS with refractory anemia; *MDS-SLD* MDS with single lineage dysplasia; *MDS-U* MDS unclassifiable; *MDS-RAEB1* MDS with refractory anemia and excess blast-1; *MDS-RAEB-2* MDS with refractory anemia and excess blast-2; *MDS-EB2* MDS with excess blast-2; (BM > 10–19% or PB 5–19%); *MDS-MLD* MDS with multilineage dysplasia; *CML* chronic myeloid leukemia; *AA* aplastic anemia

A total of 67 patients with somatic *TET2* mutations were included in our study, including 44 (65.7%) male patients and 23 (34.3%) female patients (Table S4). The median age of all the patients was 66 years (range, 24–89). The median VAF was 44.1% (range, 6.6–90.9%). Unlike germline mutations, the mutation patterns of somatic mutations are diverse, including frameshift, missense, nonsense, and other mutations. The frameshift mutation was the most common mutation (*n* = 32, 39%). A total of 82 mutation sites were detected in 67 patients; one patient had three mutation sites, and 13 patients had two mutation sites. Among the mutation sites, c.2290C > T (p. Gln764Ter) was the only recurrent site. In addition, 39 (47.6%) somatic mutation sites in our study were also retrieved from the COSMIC database (Table S5, Table S6). Of the 34 mutation sites, functional analysis was performed through Hidden Markov Models (FATHMM) values (Table S4), and most of them were reported to be pathogenic in haematological neoplasms, including AML, MDS, CMML, and lymphoma. Among the 67 patients, most had a normal karyotype (*n* = 47, 70.1%). We also found that most patients had AML (*n* = 26, 38.8%), followed by 18 (26.9%) patients with MDS. Additionally, among AML patients, most were AML-M2 and AML-M4, 14 (53.8%) and 6 (23.1%), respectively. Among the nine patients with MDS/MPN, the majority were CMML patients (*n* = 6, 66.7%) (Table S4).

### Pedigree investigation of patients with germline TET2 mutation

**Fig. 2:**
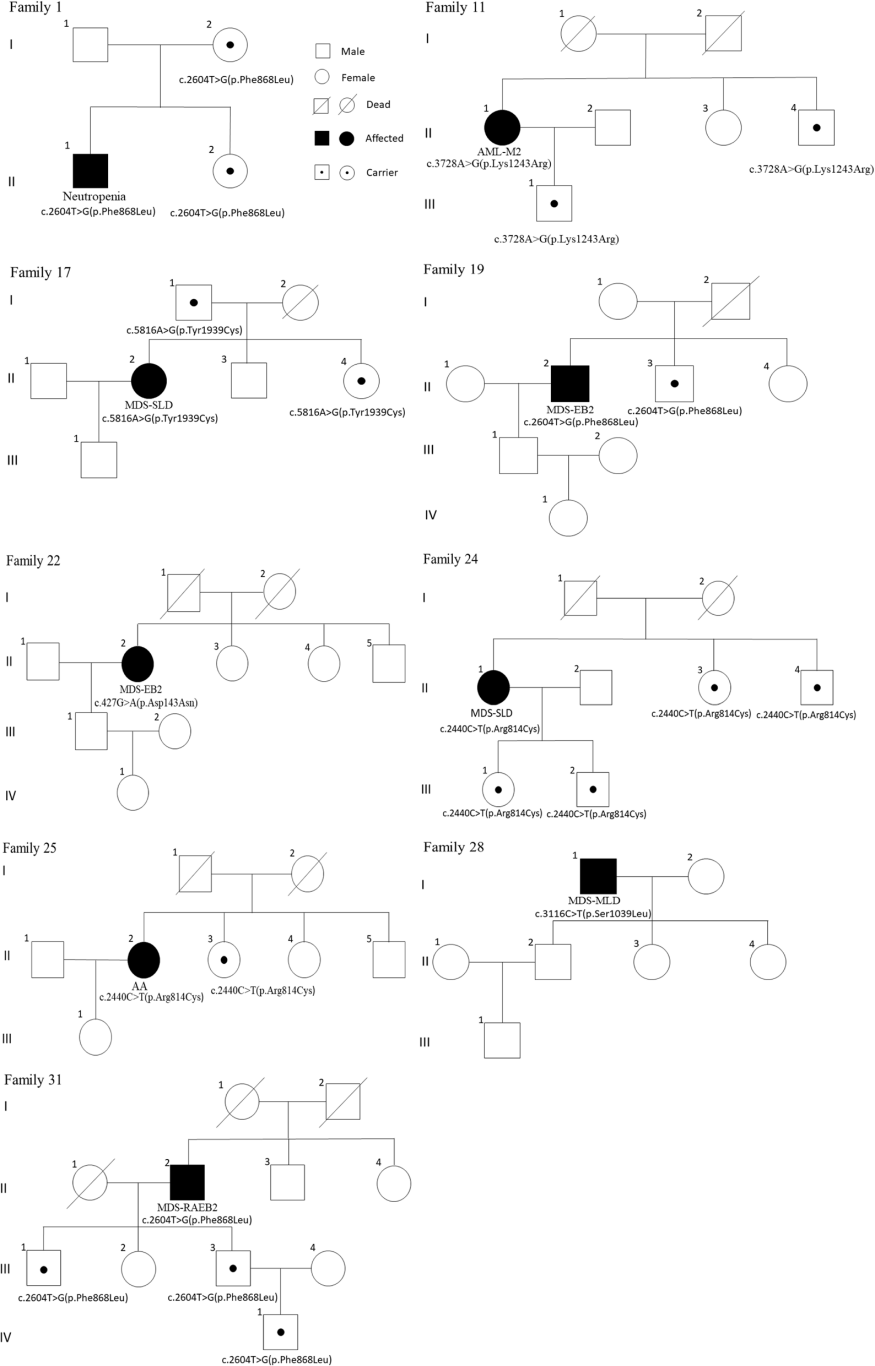
The pedigrees of nine patients with germline *TET2* mutations. Family number was in accordance with the patient number. Nails and hairs were used as the germline DNA origin to test the *TET2* status. More details of the pedigree investigation are supplied in the supplemental manuscript

Nine families accepted the detection of *TET2* status and completed the pedigree investigation. The family numbers were consistent with the patient numbers in Table 1. Among the nine patients, six patients had MDS, and one patient each had AML, AA, and neutropenia. We found that some family members carried germline *TET2* mutations, such as proband patients with the same mutation sites and types but without any haematological symptoms (Fig. 2). Moreover, the PB count test results of the two family members were normal. None of the asymptomatic carriers had any other germline or somatic gene mutations, such as proband patients (Table 2).

**Table 2:** Pedigree investigation information

Family No.	Patient No.	diagnosis	symptoms	co-mutate genes	Carriers of germline ***TET2*** mutations in lineage relatives	Same mutation or not	carriers’ symptoms	carriers’ sample	Family history
F1	P1	neutropenia	Fever	**–**	Sister (F 1-II-2) Mother (F 1-I-2)	**Y**	**–**	Hair/nail	**–**
F11	P11	AML-M2	Dizziness nasal bleeding	*ZRSR2* (GM)*KIT* (SM)	Brother (F 11-II-4) Son (F 11-III-1)	**Y**	**–**	Hair/nail/PB	**–**
F17	P17	MDS-SLD	Dizziness, fever nasal bleeding	**–**	Father (F 17-I-1) sister (F 17-II-4)	**Y**	**–**	Hair/nail	**–**
F19	P19	MDS-EB2	nasal bleeding	*TP53* (SM)	Brother (F 19-II-3)	**Y**	**–**	Hair/nail	**–**
F22	P22	MDS-EB2	–	*TP53* (SM)*ETV6* (SM)	–	**Y**	**–**	Hair/nail	**–**
F24	P24	MDS-SLD	Dizziness, fatigue	*ZRSR2* (GM) *ETV6* (SM)	Sister (F 24-II-3) Brother (F24-II-4) Daughter (F 24-III-1) Son (F 24-III-2)	**Y**	**–**	Hair/nail	**–**
F25	P25	AA	Dizziness, fatigue	*ZRSR2* (GM)	Sister (F 25-II-3)	**Y**	**–**	Hair/nail	**–**
F28	P28	MDS-MLD	Fatigue, dizziness	*NF1* (GM)	–	**Y**		Hair/nail	**–**
F31	P31	MDS-RAEB2	Dizziness, fatigue	*ZRSR2* (GM)	Sons (F 31-III-1/3) Grandson (F 31-IV-1)	**Y**	**–**	Hair/nail	**–**

Aberration: GM, germline mutation; SM, somatic mutation; PB, peripheral blood. P, patient; F, family; −, none; Y, carriers have the same mutations of germline *TET2* as proband patients

### Comparison of patients with germline TET2 mutation alone and not

Among the 33 patients, we found that 11 (33.33%) patients had germline *TET2* mutation alone, while the other 22 (66.67%) patients had other gene germline or somatic mutations. The disease distribution and bone marrow blast percentages were significantly different between the two subgroups (*P* = 0.026 and *P* = 0.004, respectively). Two of the 11 patients with germline *TET2* mutations alone had MDS, while the others were undiagnosed. Among 22 patients with other gene mutations, most were diagnosed with hematopoietic neoplasms, including 11 MDS patients and 3 AML patients. The difference in myeloblast percentages between the two subgroups was significant (*P* = 0.004). The other indicators, such as age, VAF, chromosome karyotype, haemoglobin, white blood cell count (WBC), and lymphocyte absolute count, were not significantly different (Table 3).

**Table 3:** Comparison of patients with germline *TET2* mutation alone and not

Patient’s parameters	***TET2*** GM-Alone (***n*** = 11)	***TET2*** GM-Others (***n*** = 22)	***P***
Age(Y)median(range)	46(16–74)	55.5(27–82)	0.114
Male (%)	7(63.6%)	11(50.0%)	0.712
VAF (%) median(range)	50.23(48.2–54.16)	50.70(47.5–55.0)	0.611
**Cytogenetics**			0.384
Normal or -Y alone	6(54.5%)	12(54.5%)	
Complex	0(0.0%)	2(9.1%)	
Others	1(9.1%)	5(22.7%)	
unknown	4(36.4%)	3(13.6%)	
**Diagnosis**			**0.026**
MDS/AML	2(18.2%)	14(63.6%)	
Others	9(72.8%)	8(22.7%)	
**Peripheral blood median(range)**			
Hemoglobin (g/L)	73(52–160)	83(27.0–137)	0.711
WBC (× 10^9^/L)	4.43(0.23–33.38)	4.72(1.26–13.25)	0.749
Platelet (×10^9^/L)	124(3–611)	62(8–348)	1
Absolute Neutrophils (× 10^9^/L)	1.83(0.0–25.37)	1.53(0.19–8.86)	0.711
Absolute Lymphocyte (×10^9^/L)	1.80(0.2–3.0)	1.19(0.3–2.66)	0.223
Absolute Monocyte (×10^9^/L)	0.23(0.0–0.83)	0.24(0.0–0.760)	0.863
Total Eosinophils (×10^9^/L)	0.2(0–1.0)	0.01(0–0.49)	0.065
**Bone marrow (%) median (range)**			
Myeloblast	1(0.5–4.0)	4(1.0–48)	**0.004**
Mature Lymphocyte	21(5.0–70.0)	13.25(5.5–49)	0.145
Mature Monocyte	1.5(0.5–2.0)	1.5(0.5–21.0)	0.438
Basophilic Erythroblast	1.5(0.5–2.0)	1.5(0.5–5.0)	0.71
Polychromatophilic erythroblast	9.0(3.0–17.5)	8.5(1.5–25.5)	0.76
Acidophilic Erythroblast	16.5(4–39)	15.5(2.5–51.5)	0.89

Aberration: Age (Y), Age (year); n, number of patients; *TET2* GM-Alone, *TET2* germline mutation alone; *TET2* GM-others, *TET2* germline mutation simultaneously with other gene mutations; VAF, variate allele frequency; MDS/AML, myelodysplastic syndrome/acute myeloid leukemia

A value of *P* less than 0.05 was considered statistically significant

### Comparison of germline and somatic TET2 mutations

As described previously, the VAF in patients with germline *TET2* mutations was more stable (median: 50.58%, range: 40–55%) (S1 Fig. A), and the mutation sites were not as diverse as somatic mutations (S1 Fig. B and C). We further classified and compared the comutated genes according to the functional region without a significant difference (*P* = 0.976) (Fig. S2). Of the 11 germline mutation sites identified in our study, the effect predictions of *TET2* mutations were discrepant (based on published functional studies or prediction tools), while 21/28 (75%) somatic *TET2* missense mutations were predicted to be pathogenic/likely pathogenic (Table S7).

**Fig. 3:**
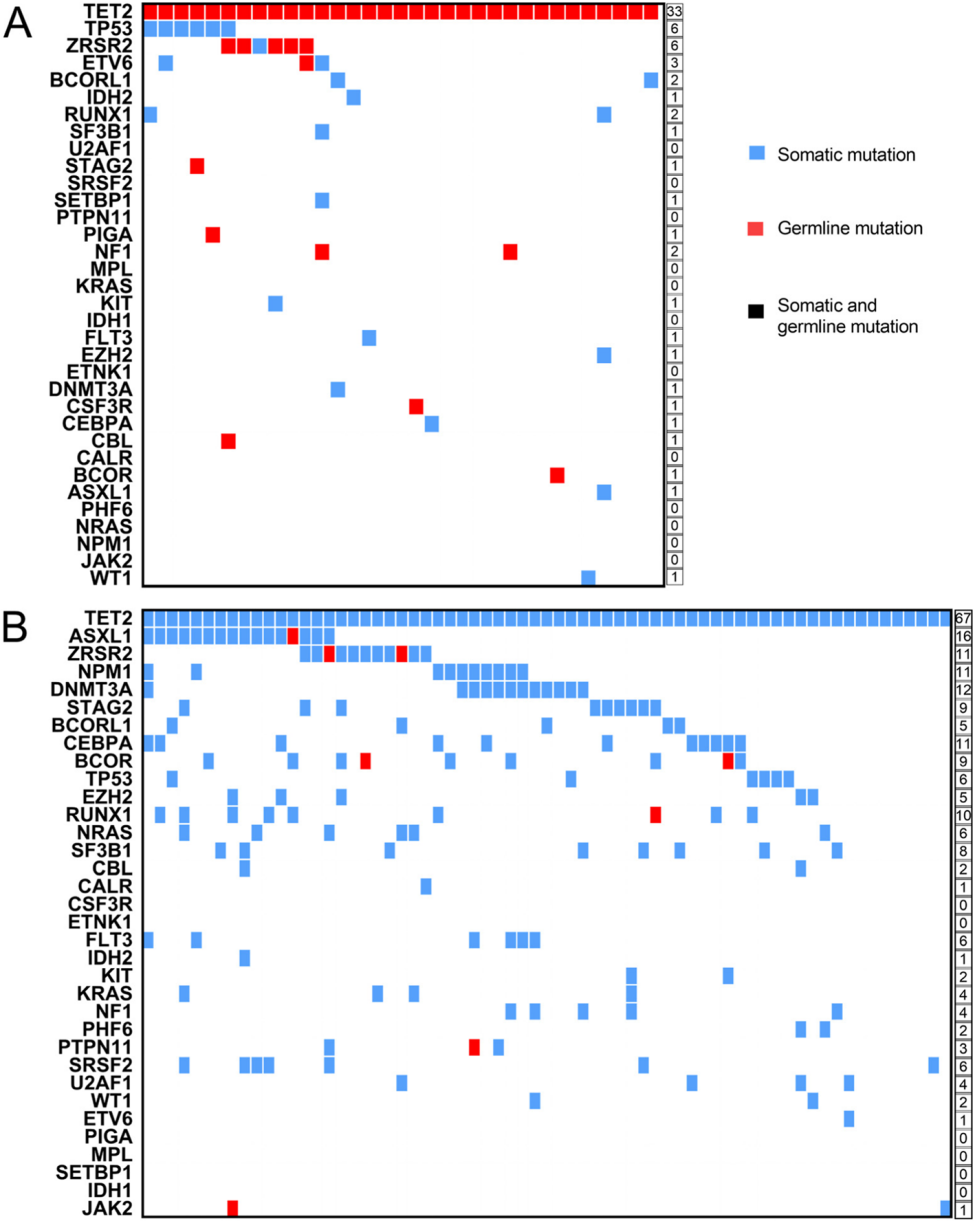
Comutated gene distribution in patients with germline and somatic *TET2* mutations. **A** and **B** Comutated genes in patients with *TET2* germline and somatic mutations, respectively. Each column in the figure represents a patient, and the digital at the right is the number of patients with each mutant gene. Germline mutation (blue), somatic mutation (red), germline and somatic mutation (black)

Among the 33 patients with germline *TET2* mutations, 11 patients had germline *TET2* mutations alone, and 22 patients also had other gene mutations. We found that the most common comutated genes with germline *TET2* mutations were germline *ZRSR2* mutations and somatic *TP53* mutations, followed by *ETV6*, BCORL1, *RUNX1*, and *NF1*. In addition, we discovered that 13 patients had only one comutated gene, including *IDH2*, *SF3B1*, *STAG2*, *SETBP1*, *PIGA*, *KIT*, *FLT3*, *EZH2*, *DNMT3A*, *CSF3R*, *CEBPA*, *CBL*, and *WT1*. Among the comutated genes, *STAG2*, *PIGA*, *CSF3R*, *CBL*, and *BCOR* were germline mutations, while *IDH2*, *SF3B1*, *SETBP1*, *KIT*, *EZH2*, *FLT3*, *CEBPA*, and *CBL* were somatic mutations. However, germline *TET2* mutations were not accompanied by mutations in the other 12 genes in our study, including *U2AF1*, *SRSF2*, *PTPN11*, *KRAS*, *MPL*, *IDH1*, *ETNK1*, *CALR*, *PHF6*, *NRAS*, *NPM1*, and *JAK2* (Fig. 3A).

Of the 67 patients with *TET2* somatic mutations, only six (9%) patients had somatic *TET2* mutations alone, while 61 (91.0%) patients had other gene mutations. Six genes did not comutate with somatic *TET2* mutations, including *CSF3R*, *ETNK1*, *PIGA*, *MPL*, *SETBP1*, and *IDH1*, whereas the other 27 gene mutations were accompanied by the somatic *TET2* mutation. Among the comutated genes, *ASXL1* was the most common comutated gene, followed by *DNMT3A*, *ZRSR2*, *NPM1*, *CEBPA*, *BCOR*, *RUNX1*, *STAG2*, *SF3B1*, *NRAS* and others. (Fig. 3B). Furthermore, only *ASXL1*, *ZRSR2*, *BCOR*, *RUNX1*, *PTPN11*, and *JAK2* had germline mutations as comutated genes.

In the 100 patients with *TET2* mutations, *MPL*, *ETNK1*, and *IDH1* gene mutations were not detected. *U2AF1*, *SRSF2*, *PTPN11*, *KRAS*, *CALR*, *PHF6*, *NRAS*, *NPM1*, and *JAK2* gene mutations were detected in patients with germline *TET2* mutations but not in patients with somatic *TET2* mutations (Fig. S3).

### Comparison of clinical features of patients with germline and somatic TET2 mutations

The average age of the patients with germline *TET2* mutations was significantly lower than that of patients with somatic *TET2* mutations [48 (range, 16–82) vs. 66 (range, 24–89), *P* = 0.0078]. There were no significant differences in sex distribution or chromosome karyotype between the two groups. Comparing the disease distribution, we found that the difference between the two groups was statistically significant (*P* = 0.0004). Among the 33 patients with germline mutations, 13 (39.4%) were MDS patients, while the 67 patients with somatic mutations were AML (*n* = 26, 38.8%), followed by 18 (26.9%) MDS patients. We further found that WBC was significantly different between the two groups [4.4(0.23–138.46) × 10^9^/L vs. 7.33 (0.97–237.29) × 10^9^/L, *P* = 0.0046]. However, the haemoglobin, platelet count, neutrophilic granulocyte count, lymphocyte absolute count, and monocyte absolute count were not significantly different (Table 4, S4 Fig). For the proportion of cells in the bone marrow, the percentage of myeloblasts in patients with somatic *TET2* mutation was higher (*P* = 0.0028); however, the percentage of mature lymphocytes, total eosinophils, and eosinophils in patients with germline mutations was higher (*P* < 0.05) (Table 4, S5 Fig). For the ten common comutated genes, only the mutation frequency of *NPM1*, *ASXL1* (*P* = 0.0144 and *P* = 0.0096, respectively), and *DNMT3A* genes were also able to indicate the difference (*P* = 0.0551) (Table 4).

**Table 4:** Clinical characteristics of patients with germline and somatic *TET2* mutations

Patient’s parameters	Statue of ***TET2*** mutation		***P***
	Germline mutation(***n*** = 33)	Somatic mutation(***n*** = 67)	
Age(Y)median(range)	48(16–82)	66(24–89)	**0.0058**
Male (%)	18(54.5%)	44(65.7%)	0.381
VAF (%) median(range)	50.6(40.5–55)	44.1(6.14–95.9)	**< 0.0001**
**Cytogenetics (%)**			0.421
Normal or -Y alone	18(54.5%)	47(70.1%)	
Complex	2(6.1%)	4(6%)	
Others	6(18.2%)	9(13.4%)	
Unknown	7(21.2%)	7(10.4%)	
**Diagnosis (n)**			**0.0004**
MDS	13(39.4%)	18(26.9%)	
AML	3(9.1%)	26(38.8%)	
AA	4(12.1%)	1(1.5%)	
MDS/MPN	0(0%)	10(13.4%)	
Others	13(39.4%)	12(19.4%)	
**Peripheral blood median(range)**			
Haemoglobin (g/L)	74(27–160)	81(37–135)	0.2613
WBC (×10^9^/L)	4.4(0.23–33.38)	7.33(0.97–237.29)	**0.0046**
Platelet (× 10^9^/L)	57.5(3–611)	59(6–1085)	0.533
Absolute Neutrophils (×10^9^/L)	1.58(0–25.37)	1.89(0.06–92.99)	0.5216
Absolute Lymphocyte (×10^9^/L)	1.33(0.2–3)	1.63(0.27–12.52)	0.0848
Absolute Monocyte (×10^9^/L)	0.24(0–0.83)	0.25(0–10.64)	0.4235
**Bone marrow (%) median(range)**			
Myeloblast	2(0.5–15.5)	7.25(0.5–86)	**0.0028**
Basophilic Erythroblast	1.5(0.5–5)	1.5(0.5–9)	0.9502
Polychromatophilic erythroblast	8.5(1.5–25.5)	6.5(0.5–36)	0.2890
Acidophilic Erythroblast	16.5(2.5–51.5)	10.75(0.5–48.5)	0.1005
Mature Monocyte	1.5(0.5–21)	3(0.5–12)	0.0799
Mature Lymphocyte	16(5–70)	6.5(0.5–42.5)	**< 0.0001**
Total Eosinophils	2(0.5–10.5)	1(0.5–9)	**0.0247**
ESG	1.5(0.5–5)	0.5(0.5–3)	**0.0083**
**Mutate gene(n)**			
*CEBPA* (+/−)	1/32(3.0%)	11/56(16.4%)	0.0973
FLT3 (+/−)	1/32(3.0%)	6/61(9.0%)	0.4202
*NPM1* (+/−)	0/33(0.00%)	11/56(16.4%)	**0.0144**
*RUNX1* (+/−)	2/31(6.1%)	10/57(14.9%)	0.3273
*ASXL1* (+/−)	1/32(3.0%)	16/51(23.9%)	**0.0096**
TP53(+/−)	6/26(18.18%)	6/61(9.0%)	0.2027
*DNMT3A* (+/−)	1/32(3.0%)	12/55(17.9%)	**0.0551**
ZRSR2 (+/−)	6/28(18.18%)	11/56(16.42%)	> 0.999
SF3B1 (+/−)	1/32(3.0%)	8/59(11.9%)	0.2646
SRSF2 (+/−)	0/33(0.0%)	6/61(9.0%)	0.1739

Aberration: Age (Y), Age (year); n, number of patients; VAF, variate allele frequency; ESG, Eosinophilic segmented granulocytes; *P* value less than 0.05 indicates statistical significance

We further selected MDS patients from the two groups of patients with germline and somatic *TET2* mutations. Eventually, we included 13 MDS patients with germline *TET2* mutations and 18 MDS patients with somatic *TET2* mutations. Comparing the characteristic indicators, we discovered that only the difference in the VAF between the two subgroups was statistically significant. The differences in age, sex, cytogenetics, peripheral blood cell count, percentage of cells in bone marrow, and comutated genes were not significant (Table S8)

### Comparison of survival of patients with germline and somatic TET2 mutations

**Fig. 4:**
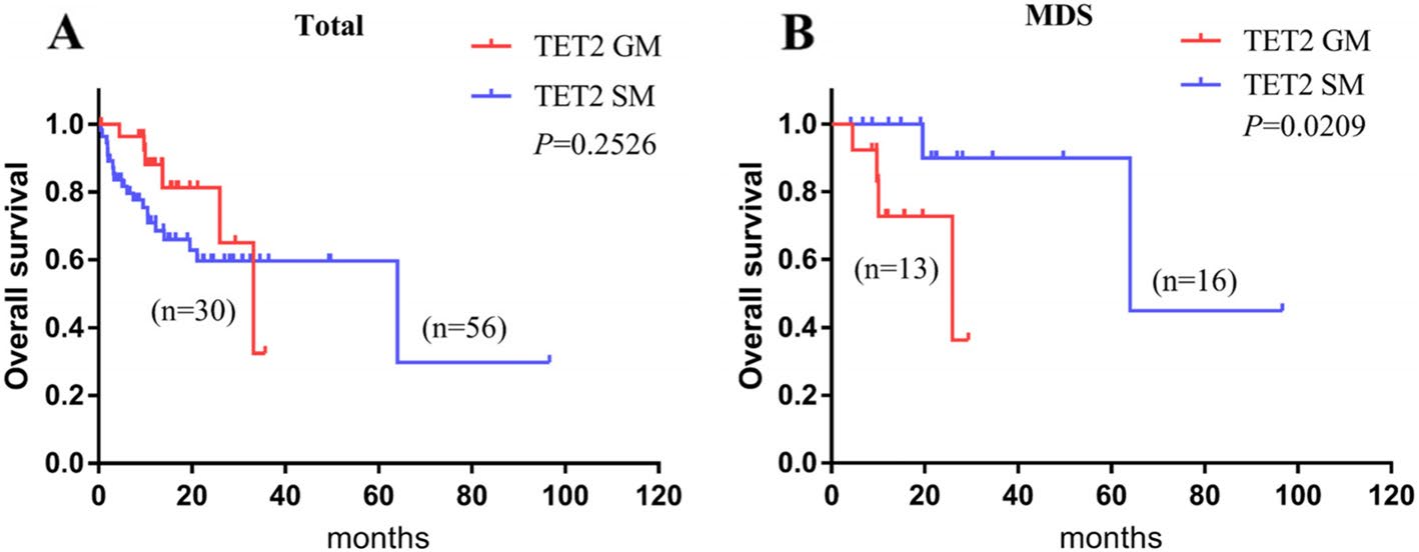
Survival outcomes in patients with germline and somatic *TET2* mutations. Kaplan–Meier curves are stratified by *TET2* mutation status: germline *TET2* mutation (red), somatic *TET2* mutation (blue). **A** OS in all patients with germline and somatic *TET2* mutations. **B** OS in MDS patients with germline and somatic *TET2* mutations

On 22 February 2020, a total of 30 patients with germline *TET2* mutations and 56 patients with somatic *TET2* mutations were included in the survival analysis. Among the 30 patients with germline mutations, six (20.0%) died, 19 (63.3%) survived, and five (16.7%) were out of contact. Among the 56 patients with somatic mutations, 21 (37.5%) died, 21 (37.5%) survived, and 14 (25%) were not accessible for follow-up. The median survival times of patients with germline and somatic *TET2* mutations were 33.3 months and 64 months, respectively. The two-year overall survival (2y-OS) was 83 and 62.7% (*P* = 0.2651) (Fig. 4A), respectively. We further selected patients without *ASXL1*, *NPM1*, and *DNMT3A* gene mutations in the two groups for sub-analysis and found no significant difference in overall survival between the two groups (Fig. S6). However, we found that the median survival time of MDS patients with germline *TET2* mutation was significantly shorter than that of MDS patients with somatic mutation (11.7 months vs. 64 months) (Fig. 4B). We further considered age (*P* = 0.666), VAF (*P* = 0.065), and IPSS-R (*P* = 0.695) as covariates and adjusted via multivariate analysis of the Cox regression model, finding that the difference in 2-y OS was not significant (95% CI: 0.9–77.5, *P* = 0.062). Furthermore, we compared the survival of patients with *TET2* mutation alone in the two groups, including 11 patients with germline *TET2* mutations and four patients with somatic *TET2* mutations. Due to no deaths in the two groups, the median survival was not available, and there was no significant difference in survival between the two groups (*P* > 0.9999).

## Discussion

Previous studies reported that pathogenicity was more likely to be present in two situations: AF was less than 0.05 and 0.01% in the 1000 Genomes and ExAC databases, respectively, or not reported in the two databases [29, 30]. However, in our study, the AF and MAF of *TET2* mutations were all less than 1% (1000 genome, ExAC, gnome AD, and gnome exome-AD) or not reported, indicating a rare mutation to exclude the likelihood of SNP [26]. All 100 patients with *TET2* mutations in our study had significant alterations in blood count to exclude the possibility of CHIP. Thirty-nine (47.6%) somatic *TET2* mutation sites in our study also exist in the COSMIC databases (S4 table, S5 table) and in the ClinVar databases, which was a powerful support for our study.

We found that most family members of proband patients with germline *TET2* mutations were asymptomatic carriers of germline *TET2* mutations by detecting gDNA from hair/nails, which indicated the possibility of family aggregation of germline *TET2* mutations. The similarity of germline *CEBPA*, *DDX41* and *RUNX1* mutations associated with haematological diseases warrants further study [7, 31, 32], such as the long time to follow-up with the family members. Among the 33 patients with germline *TET2* mutations, 11 patients only had germline *TET2* mutations, and 22 patients also had other gene mutations. Comparing the two subgroups, we discovered that patients in the former group presented with cytopenia, such as thrombocytopenia, anemia, neutropenia, and others. However, with alterations in blood count or morphology in BM, three of them met the criteria for haematological tumours, including two MDS and one CML (BCR-ABL1+). However, 14 of the other 22 patients were diagnosed with haematological tumours, mainly AML (*n* = 3) and MDS (*n* = 11). This difference indicates that germline *TET2* mutation alone may not be sufficient to initiate haematological neoplasms, and other gene comutations may be necessary for initiation. This phenomenon is in accordance with previous reports that *TET2* mutations alone often require other gene mutations as a secondary hit to induce the occurrence and development of tumours [14, 15]. This suggests that germline *TET2* mutations are predisposed to haematological tumours under the second hit of other gene mutations.

We further compared the characteristics of patients with germline and somatic *TET2* mutations. The median age of patients with germline *TET2* mutations was younger (*P* = 0.0058), which is similar to the younger age of patients with germline *CEBPA* and *RUNX1* mutations [6, 32]. However, the difference in median age between the two MDS subgroups was not significant, which may indicate that patients with germline *TET2* mutations need a long incubation period to develop MDS or other haematological tumors, such as the germline *DDX41* mutation in haematological neoplasms [7]. In the early stage, patients with germline *TET2* mutations present with single lineage cytopenia for a long time and eventually develop haematological neoplasms under the second hits of other gene mutations. We also found that most patients in the two groups had normal karyotypes, in accordance with previous conclusions [33, 34]. In terms of disease distribution, patients with germline mutations were predominantly MDS, while patients with somatic mutations were AML (*P* = 0.0004). For this result, we deduced that it may be related to the bias of the patient visits, and the short observation time was not enough to observe the progression of MDS or other diseases to AML. In fact, patients with somatic *TET2* mutations were more likely to have *ASXL1*, *NPM1*, and *DNMT3A* gene comutations, which may be another potential factor for the disease differences. In addition, we also found that the 28 AML patients with somatic *TET2* mutations were mainly M2 (*n* = 14, 53.8%) and among the nine MDS/MPN patients (66.7% CMML). We hypothesised that this may be because *TET2* impacts the differentiation of progenitor cells into myelomonocytic cells and neutrophilia, as in previous reports [35–37]. Pan et al. reported that *TET2* deletion induces mice to transform into myeloid tumours, mainly manifesting as mononucleosis and neutrophilia [35]. For other comutation genes, we discovered that *IDH1/2* mutations rarely appeared in 100 patients, which was almost mutually exclusive with the *TET2* mutation. This phenomenon is similarly reported in previous reports [38, 39].

For the peripheral blood parameters and the proportion of bone marrow cells, we discovered that WBC count and the proportion of myeloblasts were both significantly higher in patients with somatic mutations (*P* = 0.0349 and 0.0028, respectively). Considering the difference in disease distributions, we deduced that the result may be associated with it. However, it is uncertain whether the differences in WBCs and myeloblasts are affected by differences in germline and somatic *TET2* mutations because previous articles reported that AML patients with *TET2* mutations have a higher WBC count [34, 40]. We also observed that the proportion of mature bone marrow lymphocytes, total eosinophils, and eosinophils in patients with germline mutations was higher (*P* < 0.05). However, these differences were not observed in the two MDS subgroups.

The prognosis of *TET2* mutations in haematological tumours is controversial; some articles have reported that it has a prior prognosis in MDS patients or has no significant impact [17, 41], while others have reported that it has no significant influence or an adverse impact in AML patients with normal karyotypes [42, 43]. We compared survival between the two groups and found no difference in 2-y OS. However, in the MDS subgroup, the prognosis of patients (2-year OS) with germline mutations was poorer than that of patients with somatic mutations (2-year OS: 2-year OS: 72.7% vs 91.7%, HR = 5.3, 95% CI: 0.89–32.2, *P* = 0.0209). We used VAF (*P* < 0.05), age, and IPSS-R as covariates to further compare the 2-y OS between the two MDS subgroups using the Cox regression model *(P* = 0.062). Although the value of *P* was slightly greater than 0.05, it could not be denied that germline *TET2* mutation might be an independent poor prognostic factor for patients with MDS. According to IPSS-R risk stratification, 77% of patients with germline *TET2* mutations were at medium/high risk, while 61.1% of patients with somatic *TET2* mutations were at medium/high risk. We speculated that this risk difference might be a factor affecting the prognosis of patients with MDS. In addition, patients with germline *TET2* mutations may have a poor response to traditional chemotherapeutics and need more active treatment measures in the early stage, such as hematopoietic stem cell transplantation (HSCT), which is similar to germline GATA2 and *RUNX1* mutations [10, 32]. To avoid the impacts of other gene mutations on the prognosis as much as possible, we compared patients with only germline or somatic *TET2* mutations but found no difference. Therefore, whether germline and somatic *TET2* mutations have different effects on the prognosis of patients with haematological disease is still ambiguous.

Although our study is the first to report the role of germline *TET2* mutations in haematological diseases, there are still many limitations. First, the sample size in our study was small. Second, we could not completely avoid the impact of other gene mutations on clinical characteristics and prognosis. Third, we did not collect a complete family history of every proband with germline mutations and only collected nail and hair specimens from nine family members. Fourth, because the number of patients accepting demethylation drugs was small, we did not compare the response to demethylation drugs between the two groups. Finally, some patients were lost to follow-up, and the follow-up time was not sufficient to observe disease progression, so we did not analyse the prognosis between the two groups.

## Conclusions

By combining our data with public databases, we supposed that germline *TET2* mutations have a family aggregation in haematological diseases and that patients with germline *TET2* mutations may be younger. More importantly, the *TET2* gene may be a predisposition gene for haematological malignancy, initiating the tumour with the second hits from other gene mutations. Meanwhile, germline *TET2* mutations may result in a higher proportion of WBCs and myeloblasts and a lower proportion of eosinophils and lymphocytes. Moreover, we propose that germline *TET2* mutations may be an adverse factor for MDS patients. Germline gene mutations have played a more significant role in haematological diseases, especially for proper clinical management and long-term follow-up of affected individuals. Therefore, we hope that our study will provide helpful and significant information for physicians to recognise, diagnose, and manage patients with germline *TET2* mutations.

## Supplementary Information

**Additional file 1.**

**Additional file 2.**

**Additional file 3.**

**Additional file 4.**

**Additional file 5.**

**Additional file 6.**

**Additional file 7.**

## Data Availability

Most data generated or analysed during this study are included in this published article. Other data were obtained freely from public databases, including gnomAD (https://gnomad.broadinstitute.org/), ClinVar (https://www.ncbi.nlm.nih.gov/clinvar/), COSMIC (https://cancer.sanger.ac.uk/cosmic/download), and (https://cancer.sanger.ac.uk/cosmic/gene/analysis?ln=TET2).

## Abbreviations

*TET2*: Tet methylcytosine dioxygenase 2
NGS: Next generation sequencing
BM: Bone marrow
PB: Peripheral blood
VAF: Variant allele frequency
OS: Overall survival
HR: Hazard Ratio
CI: Confidential intervals
AML: Acute myeloid leukemia
MDS: Myelodysplastic syndrome
FPD/AML: Familial platelet disorder predisposition to acute myeloid leukemia
SNP: Single nucleotide polymorphism
WBC: White blood cell
HM: Haematological malignancies
WHO: World Health Organisation
HSC: Hematopoietic stem cell
CMML: Chronic myelomonocytic leukemia
MPN: Myeloproliferative neoplasms
AA: Aplastic anemia
AF: Allele frequency
MAF: Minor allele frequency
ExAC: Exome Aggregation Consortium
DAC: Decitabine
AZA: 5-azacitidine
IPSS-R: International Prognostic Scoring System revised
HSCT: Hematopoietic stem cell transplantation
