# Supplementary material for: Pedigree investigation, clinical characteristics, and prognosis analysis of haematological disease patients with germline TET2 mutation
Source: BMC Cancer. 2022 Mar 12;22:262. doi: 10.1186/s12885-022-09347-0 (PMC8917718; doi:10.1186/s12885-022-09347-0)
Supplement: Supplementary file 1 — Additional file 1. [file 12885_2022_9347_MOESM1_ESM.docx]

**Pedigree investigation, clinical characteristics and prognosis analysis of hematological disease patients with germline TET2 mutation**

**Xia Wu^1^, Jili Deng^1^, Nancheng Zhang^1^, Xiaoyan Liu^1^, Xue Zheng^1^, Tianyou Yan^1^, Yantao Ling^1^, Wu Ye^1^, Yuping Gong^*^**

**Supplementary figures and tables**

**S1 Fig.**


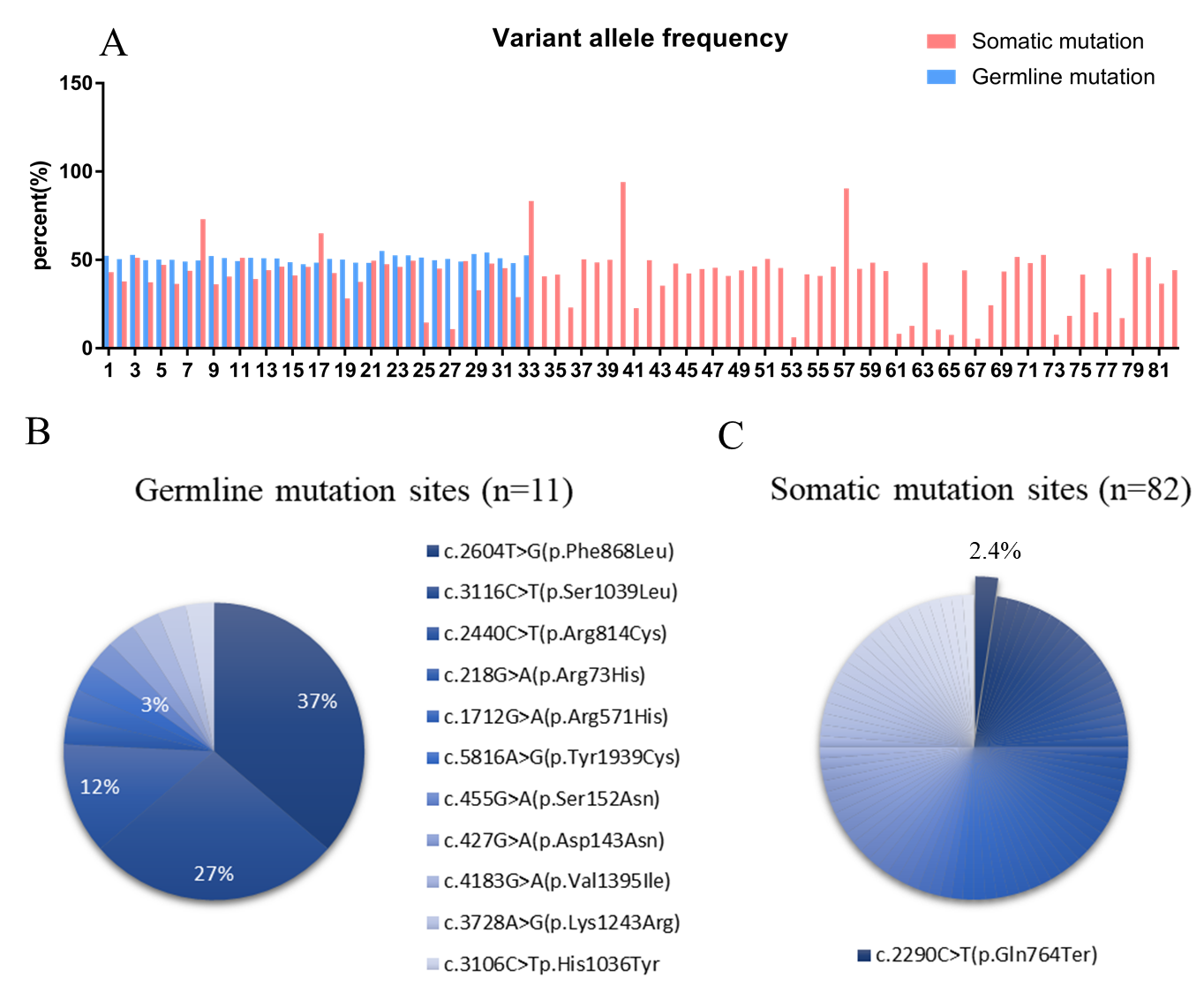


**S1 Fig. Comparison of variate allele frequency (VAF) and mutation sites between germline and somatic TET2 mutation (A)**.

The VAF distribution of germline and somatic TET2 mutation, germline mutation (orange), somatic mutation (blue) (P<0.0001). **(B)** and **(C)** The distribution of germline and somatic TET2 mutation sites, respectively. n=the number of mutation sites, 3% in **(B)** means that the remaining 8 sites only appear once, with a frequency of 3%.

**S2 Fig.**


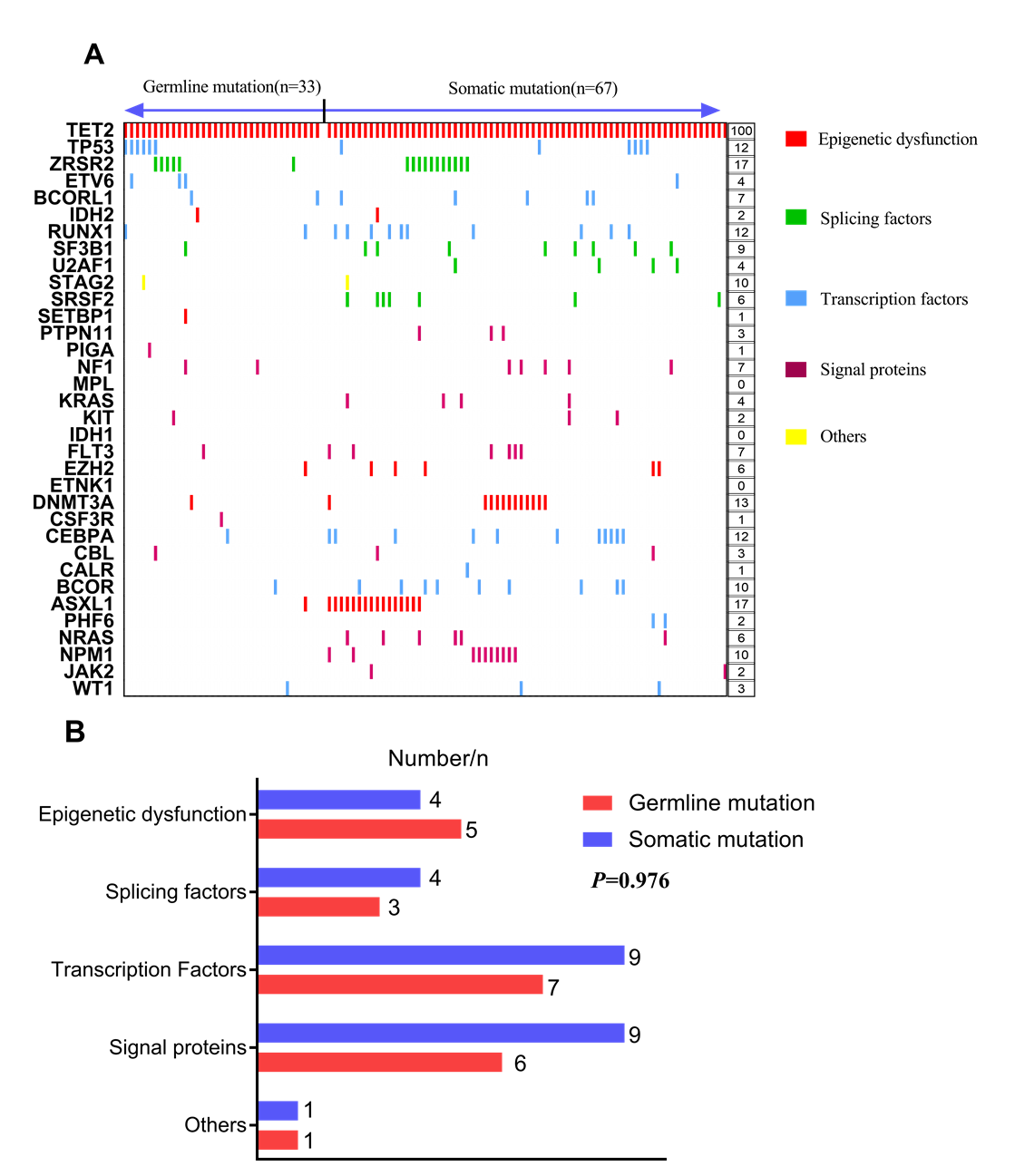


**S2 Fig. Distribution of TET2 co-mutated genes at different functional regions.**

(A) Specific distribution of the TET2 co-mutated genes in the function region. Each column in the figure represents a patient, including 33 patients with germline TET2 mutation and 70 patients with somatic TET2 mutation. Four function regions were included. Epigenetic dysfunction (red), splicing factors (green), transcription factors (blue), signal proteins (vermeil) and Others (yellow).

(B) the total number of commutated genes in each function region. Germline TET2 mutation (red), somatic TET2 mutation (dark blue).

**S3 Fig**


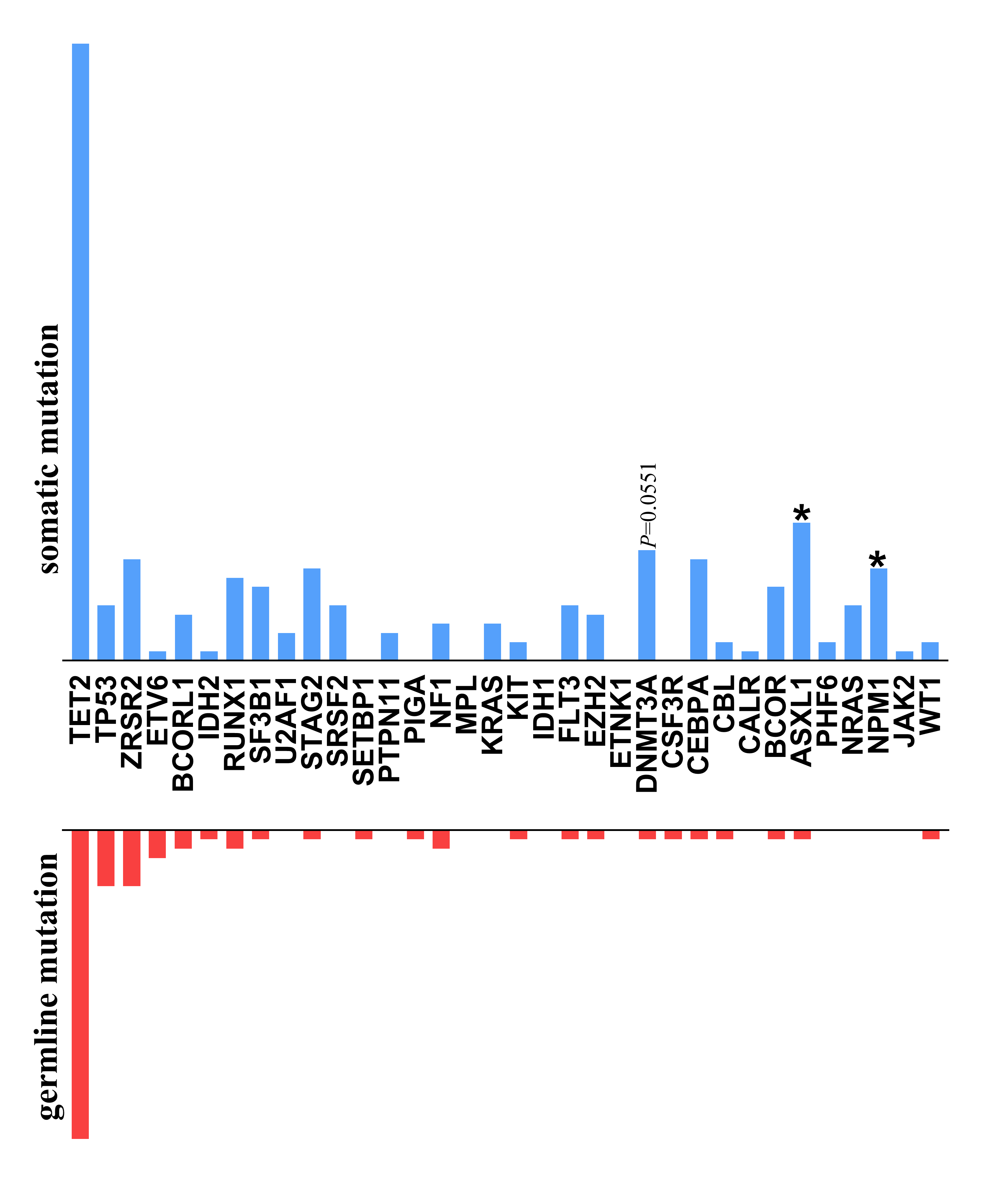


**S3 Fig. Co-occurrence and exclusivity of co-mutated gene with germline and somatic TET2 mutation.**

Somatic TET2 mutation (blue), germline TET2 mutation (red). The longer of the histogram, the more of the cases. * means this gene distribution between the two group of patients was statistic significant (*P*<0.05).

**S4 Fig**


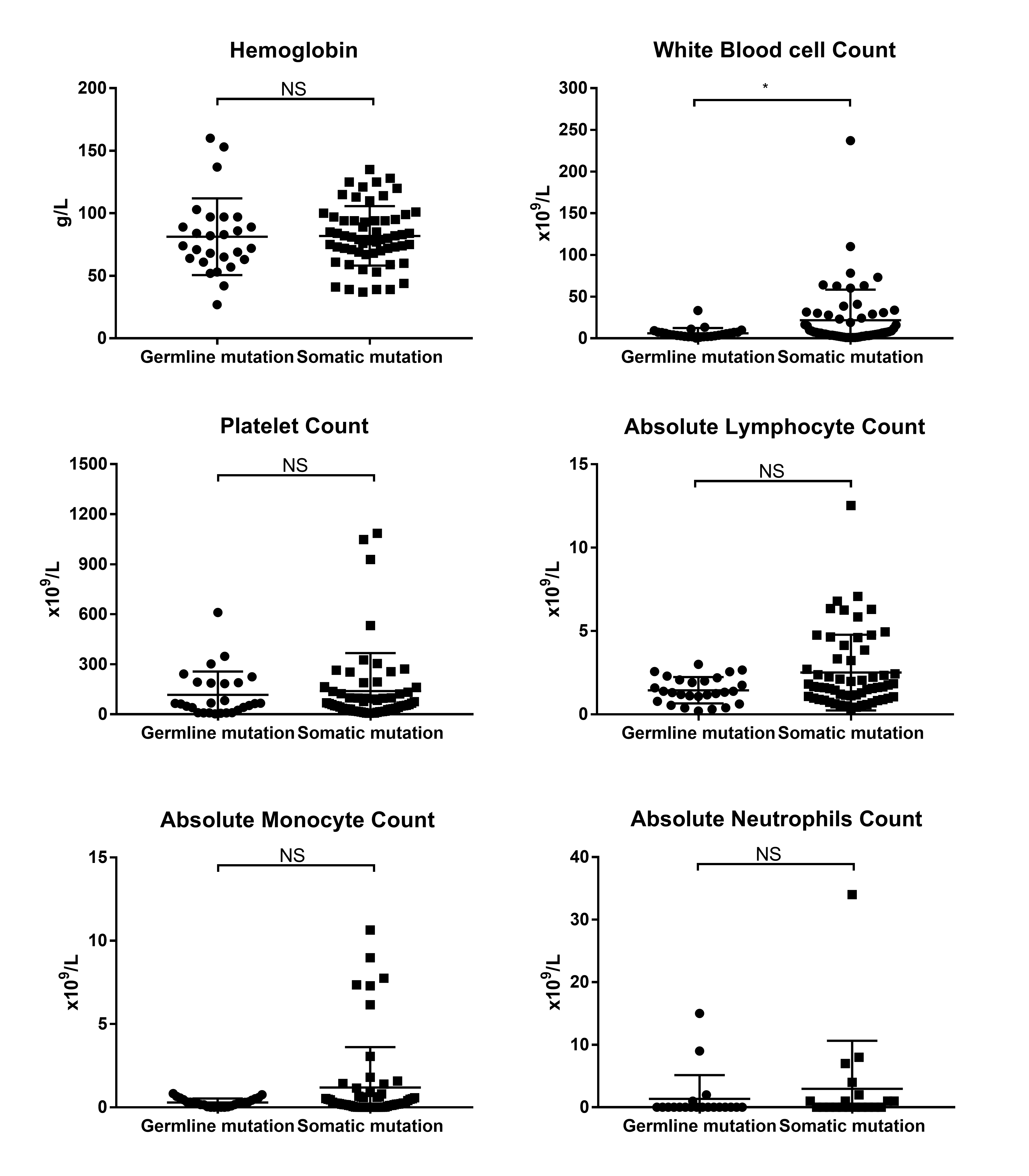


**S4 Fig. Peripheral blood parameters in patients with germline and somatic TET2 mutation.**

Note: NS, not significant; *, difference was statistical (*P*<0.05). Peripheral blood cell count is the result of a patient's first visit to the doctor. Horizontal bars indicate medians. Vertical bars indicate interquartile ranges.

**S5 Fig**


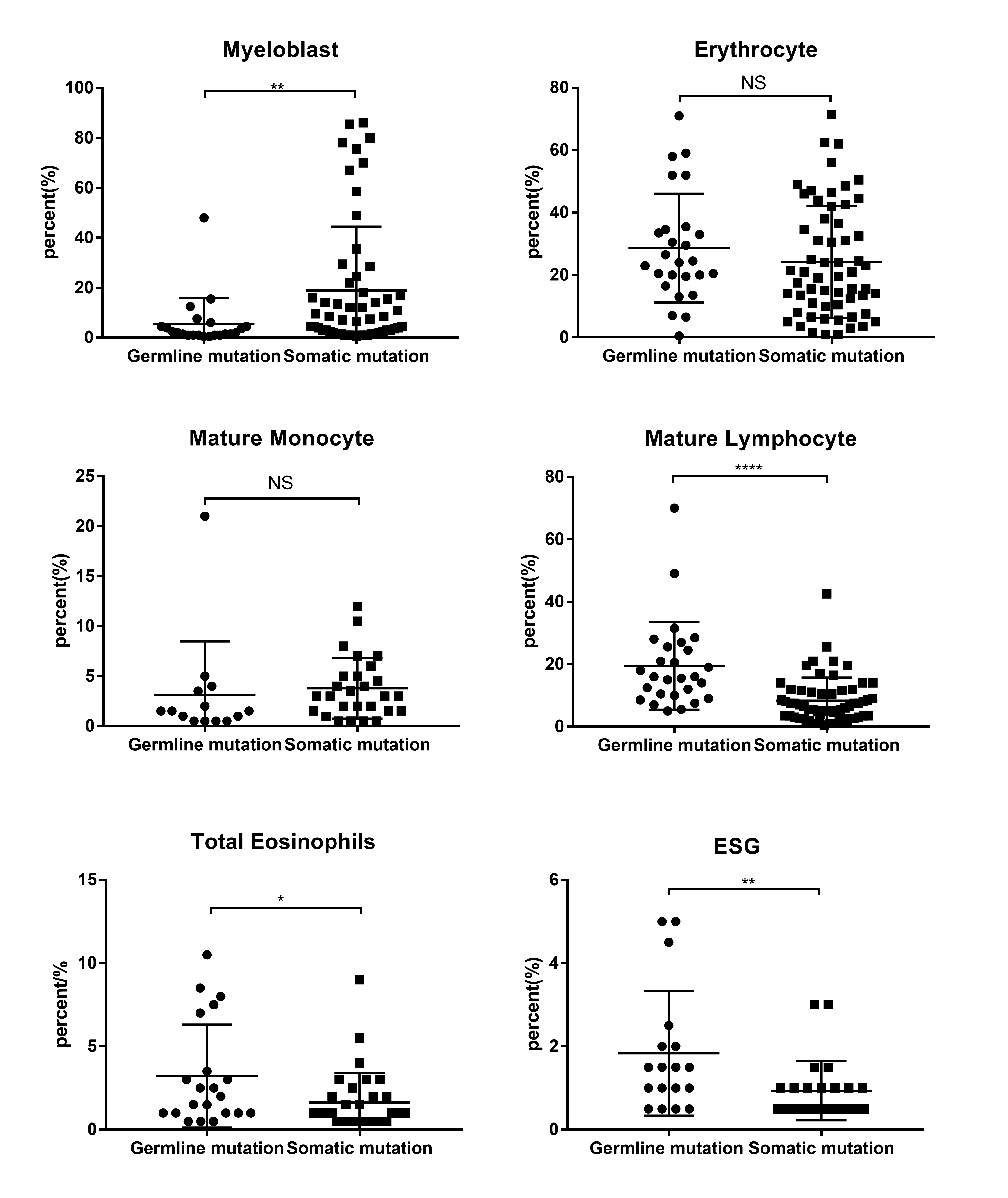


**S5 Fig. Comparison of percentage of bone marrow smear cells between patients with germline and somatic TET2 mutation.**

Note: EGS, Eosinophilic segmented granulocytes; NS, not significant; *, difference was statistical (*P*<0.05). Bone marrow smear is the result of a patient's first visit to the doctor. Horizontal bars indicate medians. Vertical bars indicate interquartile ranges. (*P<0.05，**P<0.01，***P<0.001,****P<0.0001).

**S6 Fig**


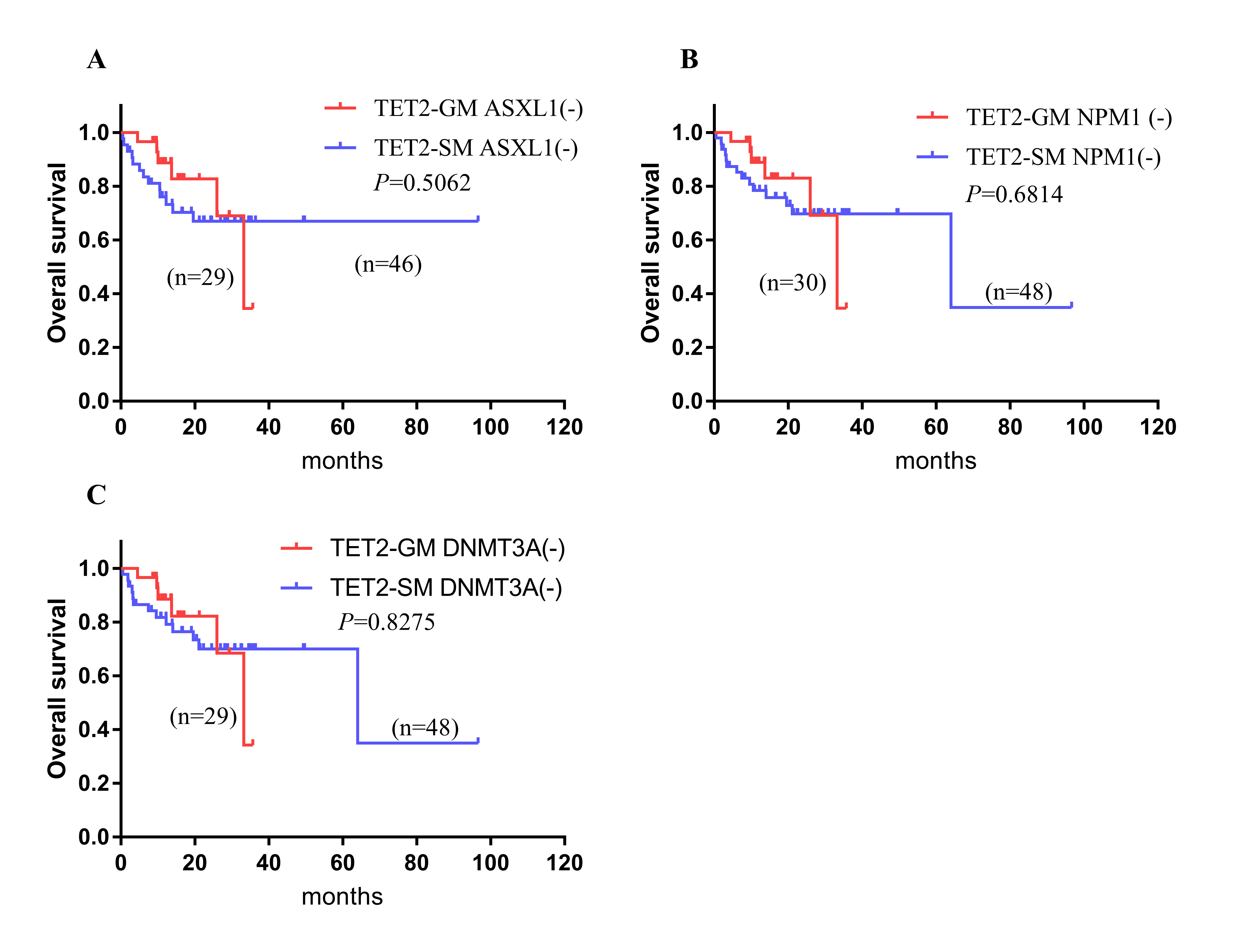


**S6 Fig. Survival outcomes in patients with germline and somatic TET2 mutation.**

Kaplan-Meier curves are stratified by TET2 mutation status: germline TET2 mutation (red), somatic TET2 mutation (blue). (A) OS in patients without ASXL1 mutation. (B) OS in patients without NPM1 mutation. (C) OS in patients without DNMT3A mutation.
