# Supplementary material for: Pedigree investigation, clinical characteristics, and prognosis analysis of haematological disease patients with germline TET2 mutation
Source: BMC Cancer. 2022 Mar 12;22:262. doi: 10.1186/s12885-022-09347-0 (PMC8917718; doi:10.1186/s12885-022-09347-0)
Supplement: Supplementary file 2 — Additional file 2. [file 12885_2022_9347_MOESM2_ESM.docx]

**S1 Table** 34 myeloid genes panel test

| **gene** | **transcript** | **gene** | **transcript** |
| --- | --- | --- | --- |
| ASXL1 | NM_015338 | MPL | NM_005373 |
| BCOR | NM_001123385 | NF1 | NM_001042492 |
| BCORL1 | NM_021946 | NPM1 | NM_002520 |
| CALR | NM_004343 | NRAS | NM_002524 |
| CBL | NM_005188 | PHF6 | NM_032458 |
| CEBPA | NM_004364 | PIGA | NM_002641 |
| CSF3R | NM_156039 | PTPN11 | NM_002834 |
| DNMT3A | NM_175629 | RUNX1 | NM_001754 |
| ETV6 | NM_001987 | SETBP1 | NM_015559 |
| ETNK1 | NM_018638 | SF3B1 | NM_012433 |
| EZH2 | NM_004456 | SRSF2 | NM_001195427 |
| FLT3 | NM_004119 | STAG2 | NM_001042749 |
| IDH1 | NM_005896 | TET2 | NM_001127208 |
| IDH2 | NM_002168 | TP53 | NM_000546 |
| JAK2 | NM_004972 | U2AF1 | NM_006758 |
| KIT | NM_000222 | WT1 | NM_024426 |
| KRAS | NM_033360 | ZRSR2 | NM_005089 |

**S7 Table** TET2 missense variants identified in this study and respective predictive values of pathogenicity from SIFT, Polyphen, PROVEAN

| **Mutation site** | **SIFT** | **PolyPhen** | **PROVEAN** |
| --- | --- | --- | --- |
| **gemline mutation** |  |  |  |
| c.2604T>G(p.Phe868Leu) | 0.018 | 0.031 | -1.35 |
| c.3116C>T(p.Ser1039Leu) | 0.001 | 0.194 | -2.28 |
| c.1712G>A(p.Arg571His) | 1 | 0 | 1.69 |
| c.2440C>T(p.Arg814Cys) | 0.002 | 0.003 | -0.57 |
| c.218G>A(p.Arg73His) | 0.255 | 0.007 | -0.87 |
| c.427G>A(p.Asp143Asn) | 0.478 | 0.001 | -0.89 |
| c.455G>A(p.Ser152Asn) | 0.103 | 0.022 | -0.62 |
| c.3728A>G(p.Lys1243Arg) | 1 | 0 | 0.36 |
| c.5816A>G(p.Tyr1939Cys) | 0.101 | 0.012 | -2.94 |
| c.4183G>A(p.Val1395Ile) | 0.004 | 0.774 | -0.92 |
| c.3106C>T(p.His1036Tyr) | 0.004 | 0 | -0.88 |
| **somatic mutation** |  |  |  |
| c.4073G>A(p.Cys1358Tyr) | 0 | 1 | -10.56 |
| c.4114A>C(p.Thr1372Pro) | 0.001 | 1 | -5.76 |
| c.3578G>A(p.Cys1193Tyr) | 0 | 1 | -10.54 |
| c.2776A>G(p.Asn926Asp) | 0.065 | 0.024 | -0.56 |
| c.4318C>T(p.Arg1440Trp) | 0.001 | 0.843 | -4.41 |
| c.3028G>A(p.Glu1010Lys) | 0.002 | 0.751 | -1.97 |
| c.3893G>A(p.Cys1298Tyr) | 0.001 | 0.998 | -9.84 |
| c.4121G>A(p.Cys1374Tyr) | 0 | 1 | -10.61 |
| c.3539T>A(p.Val1180Asp) | 0 | 0.999 | -6.58 |
| c.3781C>T(p.Arg1261Ser) | 0.001 | 1 | -5.54 |
| c.3743T>C(p.Leu1248Pro) | 0 | 1 | -6.27 |
| c.3686T>C(p.Leu1229Pro) | 0.005 | 0.998 | -5.84 |
| c.3782G>A(p.Arg1261His) | 0.001 | 1 | -4.6 |
| c.3662G>T(p.Cys1221Phe) | 0 | 0.999 | -10.56 |
| c.939del(p.Cys314ValfsTer33) | 0.028 | 0.08 | -2.54 |
| c.2959T>C(p.Cys987Arg) | 0.006 | 0.001 | -0.18 |
| c.3995T>C(p.Leu1332Pro) | 0.017 | 0.998 | -1.54 |
| c.4138C>T(p.His1380Tyr) | 0 | 1 | -5.74 |
| c.4537G>A (p.Glu1513Lys) | 0.02 | 0.321 | -1.67 |
| c.3927T>G(p.Phe1309Leu) | 0.002 | 0.986 | -5.36 |
| c.2604T>G(p.Phe868Leu) | 0.018 | 0.031 | -1.35 |
| c.5295C>G(p.Asn1765Lys) | 0.017 | 0.252 | -1.14 |
| c.5917G>A (p.Glu1973Lys) | 0.045 | 0.278 | -0.74 |
| c.4145A>G(p.His1382Arg) | 0 | 1 | -7.67 |
| c.3640C>T(p.Arg1214Trp) | 0 | 1 | -7.58 |
| c.3626T>G(p.Leu1209Arg) | 0 | 0.981 | -5.32 |
| c.4256C>G(p.Pro1419Arg) | 0.001 | 1 | -8.17 |
| c.4075C>T(p.Arg1359Cys) | 0.08 | 0.982 | -7.29 |

**Note:** SIFT value：less than 0.05 "Deleterious", Greater than or equal to 0.05 "Tolerated".

PolyPhen value: greater than 0.908 "Probably Damaging", greater than 0.446 and less than or equal to 0.908 "Possibly Damaging", less than or equal to 0.446 "Benign", unknown "Unknown".

PROVEAN value: equal to or below a predefined threshold ( -2.5) "Deleterious", greater than “-2.5” "neutral"

**S8 Table** Comparison of MDS patients with germline and somatic TET2 mutation

| **Patient's parameters** | **Statue of TET2 mutation** | | ***P*** |
| --- | --- | --- | --- |
|  | **Germline mutation**  **(n=13)** | **Somatic mutation**  **(n=18)** |  |
| Age(Y)median(range) | 63(27-75） | 50.6(24-89） | 0.465 |
| Male (%) | 8(61.5%) | 12(66.7%) | 0.532 |
| VAF (%) median(range) | 50.61(48.35-55) | 44.87(5.45-95.9) | **<0.0001** |
| **Cytogenetics** |  |  | 0.211 |
| Normal or -Y alone | 7(53.8%) | 12(66.7%) |  |
| Complex | 2(15.4%) | 0(0.0%) |  |
| Others | 4(30.8%) | 4(22.2%) |  |
| unknown | 0(0.0%) | 2(11.1%) |  |
| **Risk classification (IPSS-R)** | |  | 0.647 |
| Very low/low | 2(15.4%) | 2(11.1%) |  |
| Intermediate | 5(38.5%) | 6(33.3%) |  |
| High/Very high | 5(38.5%) | 5(27.8%) |  |
| unknown | 1(7.6%) | 5(27.8%) |  |
| **Peripheral blood median(range)** | |  |  |
| Hemoglobin (g/L) | 78(27-117) | 84(39-128) | 0.456 |
| WBC (◊10^9^/L) | 6.25(1.26-10.96) | 4.52(1.81-24.19) | 0.867 |
| Platelet (◊10^9^/L) | 67.5(9-611) | 96.0(11-928) | 0.792 |
| Ab Neutrophils (◊10^9^/L) | 2.67(0.6-5.86) | 2.44(0.51-10.64) | 0.82 |
| Ab Lymphocyte (◊10^9^/L) | 1.27(0.38-2.66) | 1.63(0.510-4.60) | 0.705 |
| Ab Monocyte (◊10^9^/L) | 0.475(0-0.83) | 0.22(0.00-1.16) | 0.106 |
| Total Eosinophils (◊10^9^/L) | 0.01(0.00-0.78) | 0.3（0.0-0.37） | 0.97 |
| **Bone marrow (%) median (range)** | |  |  |
| Myeloblast | 3.5(1-15.5) | 5.5(1.0-16.0) | 0.268 |
| Mature Lymphocyte | 12.5(5.50-31.50) | 7.5(0.5-21.0) | 0.052 |
| Mature Monocyte | 2.5(0.50-21) | 2.75(0.5-5.0) | 0.95 |
| Total Eosinophils | 2(0.5-10.5) | 1.0(0.5-4.0) | 0.055 |
| Basophilic Erythroblast | 2.0(0.50-5.0) | 2.0(0.5-4.5) | 0.999 |
| Polychromatophilic | 8.5(2.50-25.5） | 9.25(2.0-24.0) | 0.948 |
| erythrocyte |  |  |  |
| Acidophilic Erythroblast | 16.5(2.50-51.50） | 17.25(4.5-34.5) | 0.812 |
| **Mutate gene (n)** |  |  |  |
| IDH1/2 (+/-) | 1/12(7.69%) | 3/15(16.7%) | 0.6207 |
| RUNX1 (+/-) | 2/11(15.4%) | 5/14(27.8%) | 0.6672 |
| ASXL1 (+/-) | 1/12(7.69%） | 0/18(0.0%) | 0.4194 |
| TP53 (+/-) | 5/8(38.46%) | 5/14(27.8%) | 0.7007 |
| ZRSR2 (+/-) | 4/9(30.77%) | 5/13(27.78%) | >0.999 |
| SRSF2 (+/-) | 0/13(0.0%) | 4/14(22.2%) | 0.12 |
| SF3B1 (+/-) | 1/12(7.69%） | 1/17(5.89%) | >0.999 |

**Aberration:** Age (Y), Age (year); n, number of patients; VAF, variate allele frequency; IPSS-R: Revised International Prognostic Scoring System. WBC, white blood cell count.

Value of P less than 0.05 is statistic significant.
